# Disentangling how the brain is wired

**DOI:** 10.1080/19336934.2024.2440950

**Published:** 2024-12-31

**Authors:** Simon G. Sprecher

**Affiliations:** Department of Biology, University of Fribourg, Fribourg, Switzerland simon.sprecher@unifr.ch

Understanding how neural networks elicit, coordinate and modulate behaviour remains a major research focus in neuroscience. This task is anything but trivial. There are several levels of complexity that must be considered. First, the large number of neurons and the even larger number of connections. Second, there are several features, which can be used to classify neurons, such as anatomical, molecular, cellular or physiological properties. Third, the nervous system is plastic and changes during development or ageing but also dependent on previous experience or the state of the animal. This list is not exhaustive, but clearly conveys the challenges that scientists encounter when aiming to characterize neural circuits. Thus, not surprisingly, most studies typically were at some level reductionist. By focusing for instance at specific neurons or synapses it became possible to link genetic mutations to alterations in physiology, activity or behaviour. The study of the *Drosophila* neuromuscular junction is such a study system which has been widely used to provide conceptual advances of various neuronal properties. The advent of increasingly large Gal4 driver collections allow the study of sparsely labelled neuron populations down to the level of single cells. In parallel, more and more sophisticated neurogenetic effectors allowed optogenetic or thermogenetic modulation of neuronal excitability, while genetically encoded calcium or voltage sensors are critical to monitor neuronal activity. GFP-tagged proteins further allow to decipher pre- or postsynaptic organization and trans-synaptic methods even to identify circuit organization.

However, despite these advances the concept of a ‘neural circuit’ remains to a large degree detached from its embedding in the entirety of the brain. Without questioning the validity of the approach, simply looking at how two (or more) neurons or neuron types are jointly required to elicit or modulate specific behaviours remains at some level still quite reductionist. But how can we take such studies towards the circuitry of the entire brain?

Visualizing all neurons and all the synaptic connections in the brain might be the solution. Or at least a very large step towards solving the problem. On a technical level this requires a 3D electron microscopic (EM) recording of the entire brain, since all synaptic connections in a dense neuropil at that scale can only be resolved with EM – at least currently. The hurdle here remains the size of a brain- even the *Drosophila* brain. Several pioneering studies from different research group in the past years have attempted to do exactly this. Most recently the FlyWire consortium made a full 3D EM connectome dataset of an adult female *Drosophila* openly available for the community as resource [1–5]. This first complete fruit fly brain connectome was reconstructed at the synapse-level using AI-based methods and further annotated in a community effort. The dataset includes 140,000 neurons with more than 50 million synaptic connections. Naturally the first series of publications mostly focus on the major features of the brain, the type of information that the resource provides including for instance cell classes, predicted neurotransmitters as well as the link to developmental (hemi-)lineages.

Since the database is open access the *Drosophila* neuroscience community can now integrate FlyWire into research projects. It should now become possible to identify neurons of interest, their synaptic pre- and postsynaptic partners and thereby link direct evidence for physiological, genetic or behavioural functions to the connectome. The generation of a brain-wide connectome can in some way be compared with sequencing of the whole genome: It is a large and impacting source of information, which will become more relevant and complete as further analysis is performed and more knowledge about it is gained.

Since synaptic connectomes of other nervous systems have been available for some time, we may anticipate the type of advances that we will see, and we can be aware of the limitations of EM-based synaptic connectomes. Most famously the first *C. elegans* connectome was published almost 40 years ago. Other whole nervous system connectomes include the larva of the sea squirt *Ciona intestinals* or the larva of the annelid *Platynereis dumerilii*. Having a wiring diagram and knowing which synapses are present allows us to predict to some degree how neuronal activity should be transmitted within the network. This is an essential component if we want to understand the dynamics and computations that are performed in certain brain circuits. Already at this stage connectome information was pivotal for our understanding of well known brain circuits such as the mushroom body, the optic ganglia or the central complex, where at the same time a large body of genetic and behavioural experiments were performed [6–12]. Jointly with techniques like trans-Tango, to mark pre and postsynaptic partners genetically, it is now possible to bring circuit analysis to the next level and embed genetic experiments into whole-brain wiring. Challenges remain since not all forms of neuronal communication can be identified in EM-data. For instance electrical synapses as well as volume transmission are forms of neuronal signalling that are not characterized with EM. Moreover, the weight or valence of synapses depends on the transmitters as well as the type receptors, which can are also not yet present from EM-data. Lastly, the nervous system is famous for its plasticity and there is in the meantime solid evidence that synapses differ between individuals (or between sexes) but also within an individual depending on experience (learning), age or even time of day. This, however, should in no way minder the impact of the data-set available, but may provide a ground truth for assessing such phenomena. Overall it is safe to say that the advances that come with the freely accessibly FlyWire resource, its simple and intuitive web interface can indeed be a leap in the possibilities that are used to analyse the brain. It is and remains an exciting time to do neuroscience using the fruit fly as model.
